# Investigating the novel-binding site of RPA2 on Menin and predicting the effect of point mutation of Menin through protein–protein interactions

**DOI:** 10.1038/s41598-023-35599-2

**Published:** 2023-06-08

**Authors:** Gurjeet Kaur, Manisha Prajapat, Harvinder Singh, Phulen sarma, Sanjay kumar Bhadada, Nishant Shekhar, Saurabh Sharma, Shweta Sinha, Subodh kumar, Ajay Prakash, Bikash Medhi

**Affiliations:** 1 Department of Pharmacology, Postgraduate Institute of Medical Education and Research (PGIMER), Research Block B, 4th Floor, Lab No 4044, Chandigarh, 160012 India; 2 Department of Pharmacology, AIIMS, Guwahati, India; 3 Department of Endocrinology, Postgraduate Institute of Medical Education and Research, Chandigarh, India; 4 Department of Experimental Medicine and Biotechnology, PGIMER, Chandigarh, India

**Keywords:** Computational models, Computational platforms and environments, Machine learning, Protein design, Protein structure predictions, Cancer, Computational biology and bioinformatics, Endocrinology, Oncology

## Abstract

Protein–protein interactions (PPIs) play a critical role in all biological processes. Menin is tumor suppressor protein, mutated in multiple endocrine neoplasia type 1 syndrome and has been shown to interact with multiple transcription factors including (RPA2) subunit of replication protein A (RPA). RPA2, heterotrimeric protein required for DNA repair, recombination and replication. However, it’s still remains unclear the specific amino acid residues that have been involved in Menin-RPA2 interaction. Thus, accurately predicting the specific amino acid involved in interaction and effects of MEN1 mutations on biological systems is of great interests. The experimental approaches for identifying amino acids in menin-RPA2 interactions are expensive, time-consuming, and challenging. This study leverages computational tools, free energy decomposition and configurational entropy scheme to annotate the menin-RPA2 interaction and effect on menin point mutation, thereby proposing a viable model of menin-RPA2 interaction. The menin–RPA2 interaction pattern was calculated on the basis of different 3D structures of menin and RPA2 complexes, constructed using homology modeling and docking strategy, generating three best-fit models: Model 8 (− 74.89 kJ/mol), Model 28 (− 92.04 kJ/mol) and Model 9 (− 100.4 kJ/mol). The molecular dynamic (MD) was performed for 200 ns and binding free energies and energy decomposition analysis were calculated using Molecular Mechanics Poisson–Boltzmann Surface Area (MM/PBSA) in GROMACS. From binding free energy change, model 8 of Menin-RPA2 exhibited most negative binding energy of − 205.624 kJ/mol, followed by model 28 of Menin-RPA2 with − 177.382 kJ/mol. After S606F point mutation in Menin, increase of BFE (ΔG_bind_) by − 34.09 kJ/mol in Model 8 of mutant Menin-RPA2 occurs. Interestingly, we found a significant reduction of BFE (ΔG_bind_) and configurational entropy by − 97.54 kJ/mol and − 2618 kJ/mol in mutant model 28 as compared the o wild type. Collectively, this is the first study to highlight the configurational entropy of protein–protein interactions thereby strengthening the prediction of two significant important interaction sites in menin for the binding of RPA2. These predicted sites could be vulnerable for structural alternation in terms of binding free energy and configurational entropy after missense mutation in menin.

## Introduction

Menin is a 70-kDa-tumor suppressor protein encoded by MEN1 gene whose loss of function mutation causes autosomal dominant multiple endocrine neoplasia type 1 syndrome^1^, which is typically defined by the presence of tumors in at least two of the following three organs: the parathyroid, entero-pancreatic endocrine tissue, and the anterior pituitary^2^. Menin is predominantly nuclear localization protein, and plays an important role in cell proliferation, migration, gene expression, and DNA repair^3,4^. The most consistent findings reveal that menin is engaged in a complex network of interactions affecting the DNA damage repair protein FANCD2^5^. It also connects transcription factors (JunD, Smad3, NF-κB), chromatin-modifying enzymes and human histone H3K4 methyl transferase (HMT) complexes. The interaction of the 32-kDa-subunit replication protein A subunit RPA2^6^ with menin affects the DNA replication, recombination, repair and transcription.

RPA2 is part of highly conserved mammalian RPA proteins that is involved in maintaining genome stability. Further, various studies have reported RPA2 involvement in DNA repair processes as well as DNA replication process through its interaction with multiple proteins and its strong affinity for single-stranded and double-stranded DNA (7&8). The amino acid positions from 43 to 171 are mapped as the Menin-binding region of RPA2^6^, that also contains a single-stranded DNA-binding domain and the sequences that were required for RPA3 interaction^9^. This domain is believed to be indispensable for stabilizing the interaction between the RPA heterotrimer and single-stranded DNA (ssDNA). Furthermore, this domain is thought to play a role in establishing RPA-ssDNA binding polarity, with RPA1 and RPA2 oriented to the 5′ and 3′ ends.

However, the lack of significant interaction sites with known proteins has complicated the effort to elucidate the structural information about Menin or its protein-binding partners. Very few studies have investigated the different domains in Menin as probable interacting sites for RPA2^6^. But the lack of information regarding the specific interaction sites limits the understanding of RPA2-menin interactions and their corresponding downstream effects. Interestingly, few studies have reported the high expression of RPA2 in multiple cancer types^10^, which revealed that RPA2 binds competitively with menin resulting in the inhibition of menin-NF-κB interaction. Further the oncogenic function of RPA2 was, at least in part, mediated by mitigating the antagonistic function of menin on NF-κB-mediated transactivation. Thus, the interaction of menin and RPA2 can inhibit the activation of the NF-κB signaling pathway. Recently, novel DNA damage-induced phosphorylation site at Thr-98 site of RPA2 has been identified^11^. Alternations at this site lead to ineffective DNA repair response. Thus, identifying specific amino acids sequences corresponding to specific interaction sites that harbor the effective binding of Menin with RPA2 could help in discovering potential drug candidates. Our work carries on the previously identified novel mutation in *MEN1* gene^12^ such as S606F which results in the disruption of menin-regulator interacting sites that result in the presentation of multiple endocrine tumors such as a parathyroid tumor, pancreatic endocrine tumor, and pituitary adenoma. Molecular dynamics simulations are the central approach to study point mutation-induced changes in protein structure, function, and interaction at the atomic level^13^. A combined classical molecular dynamics and molecular mechanics study can help elucidate the structural and conformational perturbation of the Menin-RPA2 interaction. In order to determine the relative efficiency of the interactions between menin-RPA2 as well as identification of specific key amino acids, the combined protein–protein docking approach, classical molecular dynamics (MD) simulations, and Molecular mechanics Poisson − Boltzmann surface area (MM/PBSA) based energy decomposition analysis was used. Further, dissecting the configurational entropy changes of the Menin-RPA2 complex during 200 ns was also employed to calculate the changes in entropy fluctuations. In addition, we have also examined the impact of mutation p.S606F in menin on the interaction changes with RPA2. Further, this study has thoroughly elucidated MD simulation-based prediction of two important RPA2 binding sites in menin, which could be vulnerable to structural alternation in terms of binding free energy and configurational entropy after missense mutation, thus highlighting the key amino acids involved in stable menin-RPA2 interactions.

## Materials and methods

All the computational jobs were carried out on HP desktop (OS: LINUX Ubuntu 18.04.02 LTS).

### Protein homology modeling, preprocessing and validation

The reference sequence of menin (*Homo sapiens*) was retrived from RefSeq database and subjected for homology modeling using swiss model server (https://swissmodel.expasy.org/)^14^. The modeled structure was validated using Maestro's PROCHECK module, and another "Protein Preparation Wizard" was used for protein preparation, including adding hydrogen, filling in missing side chains and loops, removing water beyond 5 Å. Preprocessed structure was minimized using OPLS3 forcefield at the pH 7.0. Another protein structure of RPA2 protein was retrieved from Protein data bank (PDB: 2PI2). PDB: 2PI2 contains the two different PDB of RPA14 and RPA2. For this study, we have selected the A chain of RPA2. The A-chain of RPA32 was preprocessed and minimized in a similar manner to menin.

### Validation of the target protein

The structures of menin and RPA2 were validated with the PROCHECK tool, through SAVES V5.0 server (https://saves.mbi.ucla.edu/)^15^. The PROCHECK tool helps to differentiate good and bad quality of protein structures. The stereo chemical efficiency and geometry of “residues by residues” or whole residues were analyzed by PROCHECK. The quality of selected protein models (MENIN and RPA2) was also evaluated by using the Ramachandran plot. While evaluating, we found Swiss model 3D structure and PDB 2PI2 were within an acceptable quality range and were used as models for further study. The modeled protein structure was validated using MD simulation in the apo form using a similar parameter as described in the MD simulation section.

### Protein–Protein interaction (PPIs) predictions

For the investigation of the PPIs of Menin and RPA2 protein, we have evaluated the Protein–Protein docking and binding free energy of the complex.

#### Protein–protein docking

To evaluate the protein–protein complex interactions protein–protein rigid docking approach was employed using the PIPER module of Schrodinger maestro. Briefly, menin was selected as the receptor and RPA2 as the ligand, and the number of ligand rotations to the probe box (1 Å) was set at 70,000 (maximum), which corresponds approximately to sampling every 5° in the space of Euler angles. The step size of the translational grid was 1 Å. The resulting poses were ranked by the size of the cluster from the top 1000 rigid ligand docking results. Maximum cluster generation was set up at 30 in our case; hence top 30 docked poses were produced.

#### Binding free energy

The binding free energy in form of delta G energy was evaluated using PRODIGY software (https://wenmr.science.uu.nl/prodigy/)^16^. Delta G energy was basically the binding free energy calculation for different docking poses in order to top three conformations. The PRODIGY tool evaluates the binding affinity of protein–protein interaction, which is based solely on structural properties. Kastritis et al. (2011) have shown the number of interfacial contacts (ICs) of a protein–protein complex, which also correlates with the experimental binding affinity. With the help of this tool, we have identified PPIs binding free energy of all possible RPA2-Menin interaction structures, and the top three models were chosen for confirmation based on maximum negative free energy.

### Molecular dynamic simulations study

The molecular dynamic (MD) simulations of Receptor-ligand (Menin-RPA2) complexes were performed using GROMACS^17,18^. Protein topology was prepared using CHARM27 force field and TIP3P water model was employed to solvate the protein–protein (P–P) complex system using cubic box enclosing the edge length of 10 Å. Neutralization of the P–P complex was done by adding the counter-ions in the form of NaCl. For the minimizations, periodic boundary conditions (PBC) were considered while; five thousand successive steps of energy minimization were performed using the GROMACS mdrun module. The heating of the system from 0 to 300 K for 100 ps in an NVT ensemble that indicates the velocities of the particles was adjusted to gradually increase the temperature of the system from up to 300 K while keeping the number of atoms and volume of the system constant and then the system was stabilized using a constant pressure of 1 bar for 100 ps with a time step of 2 fs per step. After the stabilization, MD simulations for 200 ns (ns) were performed. MD simulation trajectory coordinates were recentered and rewrapped for Menin (centered) with the gromacs module *trjconv* (removal of the periodic boundaries conditions) followed by visualization and analysis using visual molecular dynamics (VMD)^19^. The Cα-RMSD was calculated using the RMSD trajectory tool of VMD by employing 1st frame of the simulation structure as a reference structure.

### Binding free energy (BFE) calculation and energy decomposition analysis (EDA)

The model exhibiting least deviation in 200 ns classical MD simulation, trajectories (200 frames) was subjected to the MM/PBSA-based BFE calculation using *g_mmpbsa* tool^17^. Equation for binding free energy calculation as following:

1 $$ {\text{G}}_{{\text{x}}} = \left( {{\text{E}}_{{{\text{MM}}}} } \right) - {\text{TS}} + \left( {{\text{G}}_{{{\text{solvation}}}} } \right), $$

where x is the ligand or the protein or ligand–protein complex, G_solvation_ is the energy of solvation; E_MM_ is the average molecular mechanics potential energy in a vacuum; TS is the configuration entropy (contribution of entropy Temp. and S entropy). BFE calculations were followed by the EDA. EDA was performed to measure the contribution of each and every amino acid residue of the complex in the binding free energy. It was measured using *g_mmpbsa* tool with the python script *MmPbSaDecomp.py.*

2 $$ \Delta {\text{R}}_{{\text{x}}}^{{{\text{BE}}}} = \sum \left( {{\text{A}}_{{\text{i}}}^{{{\text{bound}}}} - {\text{ A}}_{{\text{i}}}^{{{\text{free}}}} } \right){\text{ for i }} = \, 0{\text{ to n}}. $$

where, A_i_ complex and A_i_ free are the energy of ith atom from x residue in bound and unbound forms respectively, and n is the total number of atoms in the residue.

### Configurational entropy calculations

The configurational entropy per Ca atom of different models of Menin-RPA2 complexes (Wild and Mutant) was calculated using Schlitter’s method^20^. The absolute entropy S was approximated according to Schlitter’s formula, as follows:

3 $$ S_{abs} < \, S = \raise.5ex\hbox{$\scriptstyle 1$}\kern-.1em/ \kern-.15em\lower.25ex\hbox{$\scriptstyle 2$} \, k_{B} {\text{ln det }}\left[ {{1 } + k_{B} Te^{{2}} M\sigma } \right]h^{2} $$

Here, k_B_ is Boltzmann’s constant; *h* is Planck’s constant reduced by 2π; *T* is the absolute temperature; e is the Euler value; M is the diagonal mass matrix of rank 3*N*; and *σ* is the covariance matrix of the atomic positional fluctuations.

## Results

### Protein homology modeling, preprocessing, and validation

#### Menin protein

The Fasta Sequence of menin protein retrieved from Uniport (O00255) to generate 3D structural model by swiss model (https://swissmodel.expasy.org/). The calculated GMQE value was 0.76 and QMEAN value was − 2.76. The PROCHECK tool was used for the validation. In the validation of Menin, the Ramachandran plot (Supplementary Fig. S1 and Supplementary information Annexure S1) showed that 86.8% core of amino acid residues were in the most favored region and 10.4% were in the allowed region. Over all G factor was calculated to be -0.14, the maximum deviation was 6.2 with planar groups of 92.8% falling within limits. The data is shown in Table 1.

**Table 1:** The validation of Menin protein using PROCHECK tool.

Total residues of menin protein	612
Ramachandran plot	Residues in most favourable region: 86.9% core Residues in additional allowed region: 10.4% allow Residues in generously allowed region: 1.9% gener Residues in disallowed region: 0.8%
Residues properties	Maximum deviation: 0.2 Bed contact: 0 Bond length/angle: 9.8
G factor	Dihedrals: − 0.23 Covalent: − 0.04 Overall: − 0.14
Planer Group	98.8% with a limit 72% highlighted

### RPA2 protein

RPA2 crystal structure was retrieved from the Protein data bank (PDB: 2PI2). PDB: 2PI2 contains the two different PDB of RPA14 and RPA2. For this study, we have selected the A chain of RPA2 as its distinctively involved in binding with Menin. For the validation of RPA2, the Ramachandran plot showed that 89.5% core of amino acid residues were in the most favored region and 9.7% were in the allowed region. The overall G factor was calculated to be − 0.03; the maximum deviation was 19.2 with planar groups of 100% falling within limits as shown in Supplementary Fig. S2 and Supplementary information Annexure S1.

### Protein–protein docking and binding energy

**Figure 1:**
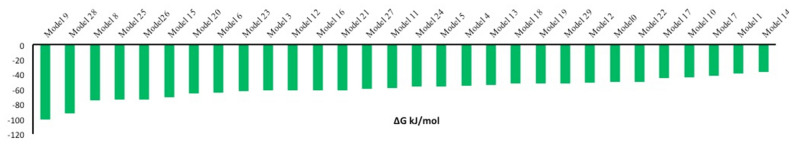
The delta G energy of different complexes (protein–protein docked models).

**Figure 2:**
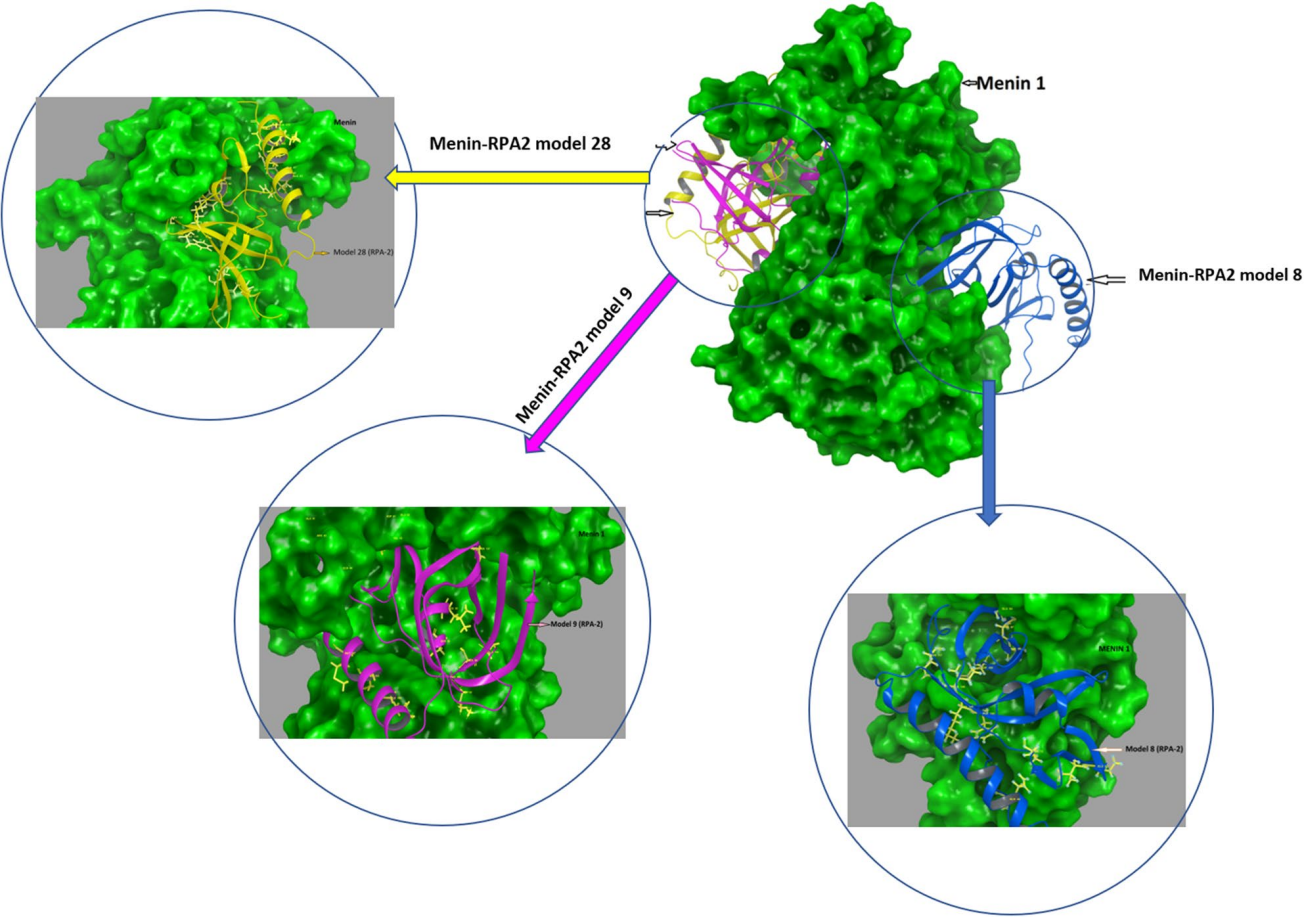
Menin interaction with RPA2 at 3 different positions. Here blue colored secondary structure of RPA-2 interacting with Menin representing as a model 8, pink colored secondary structure interacting with Menin representing the model 9 and yellow colored secondary structure of RPA-2 interacting with Menin is representing model 28.

The in-silico constructed proteins using Schrodinger maestro version 2020-3 software was used for protein–protein docking studies. A total of thirty models were generated with different binding pose sites (Fig. 1). We have also identified the top interacting amino acids, which were shown with higher interaction between menin and RPA2. We calculated the binding energy and delta G energy of all 30 conformations of the menin-RPA2 complexes using the Prodigy tool. Among all 30 different models, only 3 complexes (model 8, 28 and 9) were selected for further study on the basis of the highest delta G energy which is − 74.89 kJ/mol, − 92.04 kJ/mol, − 100.4 kJ/mol respectively. The selected models based on energy were shown in Fig. 2.

### Molecular dynamic simulation

**Figure 3:**
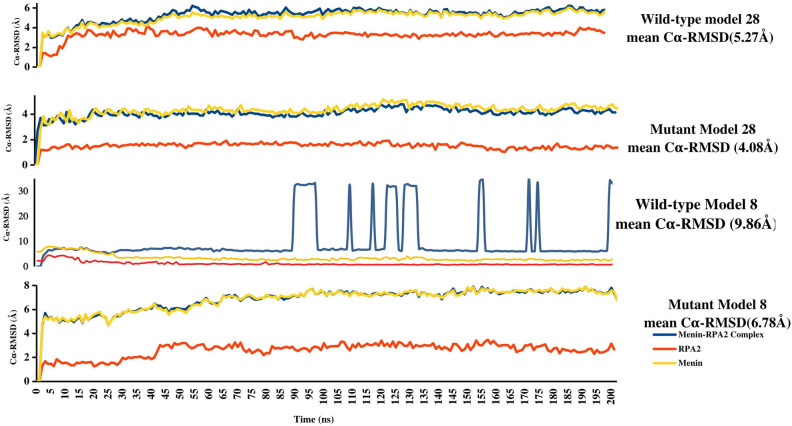
The comparative analysis of Cα-RMSD of three different model complex of Menin receptor protein-RPA-2 ligand protein and S606F mutated Menin receptor and wild type protein-RPA-2 ligand protein. (Protein viz contribution to Cα-RMSD and convergence from wild type to mutated models).

The molecular dynamic simulation study was performed for the selected protein–protein interactions complex (model 8, model 9 and model 28). The MD simulations were performed for 200 ns in this present study. The Cα-RMSD typically explained the conformations attained during the long run of MD simulation of 200 ns as in apo form (individual protein i.e., RPA2 and Menin) and P–P complex for different docked structures (Model 8, Model 9 and Model 30). The Cα-RMSD of Model 8 of Menin-RPA2 were: 5.82 Å; model 9 of Menin-RPA2 were 6.52 Å and model 28 of Menin-RPA2 were 5.27 Å. Most of the deviation is accounted for by the modeled protein menin in Apo form and P–P complex. Maximum fluctuations in Cα-RMSD in the Menin-RPA2 complex of Model 8 were contributed by the complete disassociation of P–P complex at the time point of 89thns–96thns and 120thns–130thns, therefore the binding mode of RPA2-Menin is not favored. Among these models, model 28 showed the lowest Cα-RMSD result, and there was no complex disassociation seen during the long run of MD simulation Fig. 3. Hence Cα-RMSD was majorly contributed by the modeled protein Menin and depicted the favored binding mode as shown in Fig. 3. Model 9 showed a similar binding mode towards the Menin but there was slightly more deviation (6.52 Å) found in the comparison of model 28 (5.27 Å). We have also analyzed all three different model complex structures (model 8, model 9, and model 28) and inferred that model 9 and model 28 present similar binding sites.

### Binding free energy (BFE) calculation and energy decomposition analysis (EDA)

The affinity or efficiency of Menin-RPA2 PPi was revealed by the BFE calculation. g_mmpbsa is an efficient tool that provides evidence-based support to select the Menin-RPA2 model. After the analysis, model 8 of Menin-RPA2 exhibited the most negative binding free energy of − 205.624 kJ/mol, followed by model 28 of Menin-RPA2 with − 177.382 kJ/mol. However, the Menin-RPA9 model showed a positive value of binding energy 223.018 kJ/mol, therefore model 9 was excluded from mutation analysis. So, based on BFE and EDA results, model 8 and model 28 of Menin-RPA2 were further selected for mutation study for specific Menin mutation (S606F).

### Effect of S606F on menin-RPA2 interactions

From a previous study, we have identified a frequently occurring mutation in Menin (p. S606F) in parathyroid adenoma^12^. In this context, we have evaluated the detailed effect of S606F mutation in Menin on the menin-RPA2 interaction. For this analysis, the Model-28 and 8 (Menin-RPA2 interaction complex) has been used as a wild type structure for S606F mutated Menin. We have constructed an exclusive model (Model 8 and Model 28) for mutant Menin having missense mutation on Serine 606 position altered with phenylalanine amino acid. Mutated models 8 and 28 again submitted for MD simulations, followed by superimposition of both wild type and mutant Menin. The Cα-RMSD of model 8 and model 28 of mutant Menin-RPA2 were measured to be 6.78 Å and 4.08 Å. In comparison with wild type Menin-RPA2, Cα-RMSD were found to be increased by approximately 1.0 Å for model 8 whereas approximately 1.2 Å decrease in case of model 28 of Menin-RPA2. (Fig. 3). Mutated model 28 shown clear convergence from wild type model 28 during 200 ns time frame. Hence forth, stabilization of complex is more favored by the single residue mutation at S606F.

### Binding free energy of mutant structure

**Figure 4:**
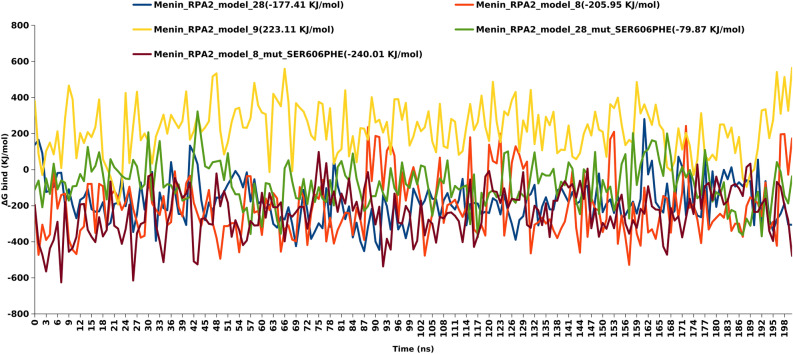
BFE (ΔG_bind) calculation for 200 frames from respective 200 ns simulated trajectories. Data are shown as Mean ΔG_binding.

In model 28 of mutant Menin-RPA2, there was a significant reduction of BFE (ΔG_bind_) by −97.54 kJ/mol in comparison with wild type Model 28 of Menin-RPA2. However, we found a slight increase of BFE (ΔG_bind_) by − 34.09 kJ/mol in Model 8 of mutant Menin-RPA2 as compared to its corresponding wild type model. Similar trend was found in BFE calculation in both mutated model 8 of Menin-RPA2 (− 240.040 kJ/mol) and mutated model 28 of Menin-RPA2 (− 79.790 kJ/mol). The comparative analysis of BFE (ΔG_bind_) of different models of wild type and mutant Menin-RPA2 interactions were shown in Table 2. The comparative trajectories of BFE (ΔG_bind_) at 200 ns were shown in Fig. 4. Interestingly S606F in Menin, plays the vital role in reduction/increase of average RMSD and BFE.

**Table 2:** Binding free energy (BFE) calculation using g_mmpbsa tool.

Models	Van der Waal energy (kJ/mol)	Electrostattic energy (kJ/mol)	Polar solvation energy (kJ/mol)	SASA energy (kJ/mol)	Binding energy (kJ/mol)
RPA2_menin Model 8	− 353.529 ± 10.412	− 1349.537 ± 40.152	1553.497 ± 43.766	− 55.167 ± 1.584	− 205.624 ± 12.215
RPA2_menin Model28	− 535.610 ± 3.505	− 856.557 ± 9.677	1290.803 ± 13.395	− 75.780 ± 0.435	− 177.382 ± 8.583
RPA2_menin Model9	− 558.575 ± 5.270	− 404.754 ± 10.653	1264.022 ± 15.190	− 77.573 ± 0.617	223.018 ± 9.929
S606F mutation					
Mut_RPA2_menin Model 8	− 339.500 ± 2.222	− 886.614 ± 9.259	1036.546 ± 10.594	− 50.276 ± 0.316	− 240.040 ± 9.474
Mut_RPA2_menin Model28	− 552.380 ± 4.404	− 763.827 ± 11.155	1312.262 ± 12.183	− 76.414 ± 0.443	− 79.790 ± 8.385

Energy kJ/mol ± sd error.

### Protein–protein interaction (PPI) analysis of wild type and mutant Menin-RPA2 complex based on energy decomposition analysis (EDA)

**Figure 5:**
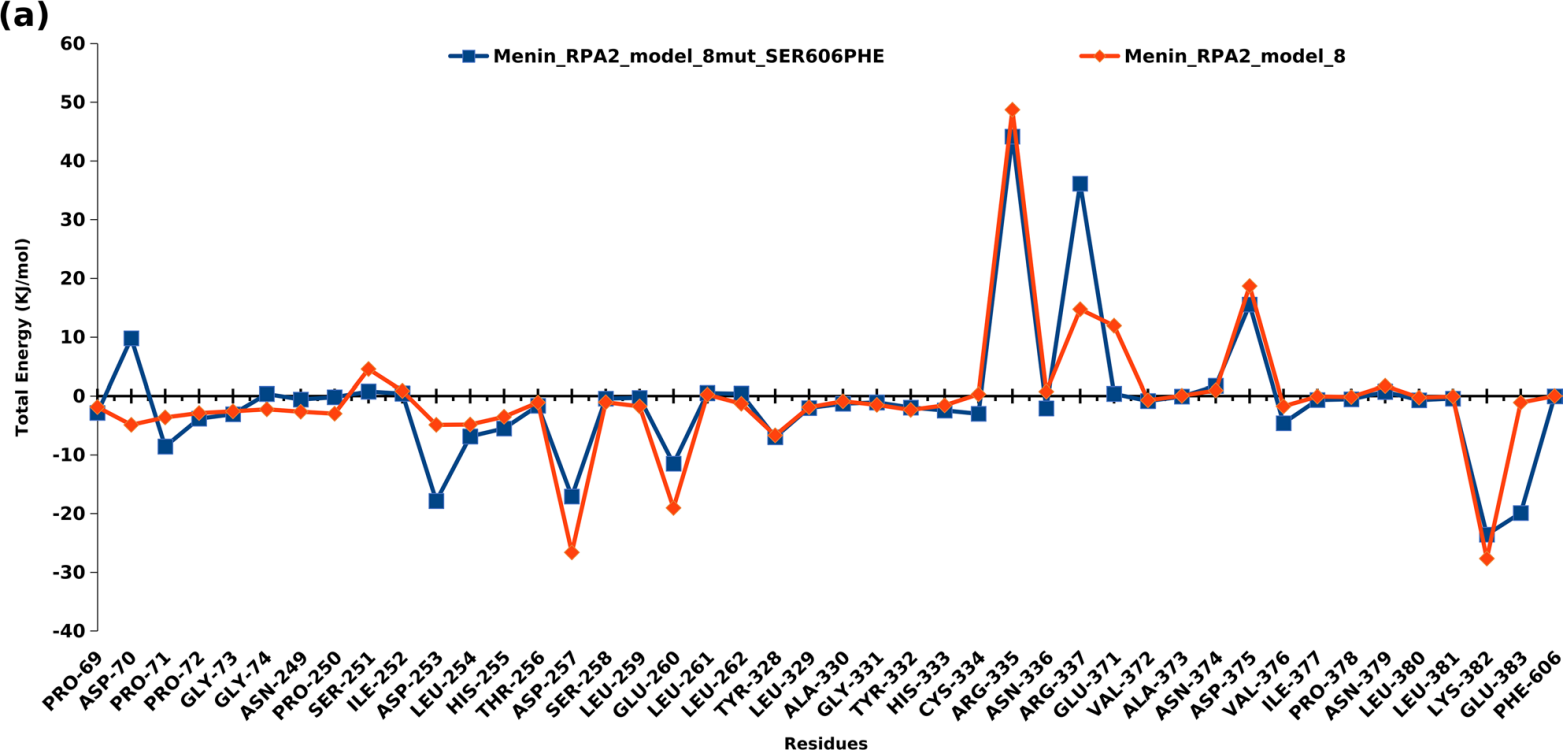
Energy decomposition analysis: depiction of per amino acid contribution to the total binding energy of wild and mutant type model 8 of Menin-RPA2.

**Figure 6:**
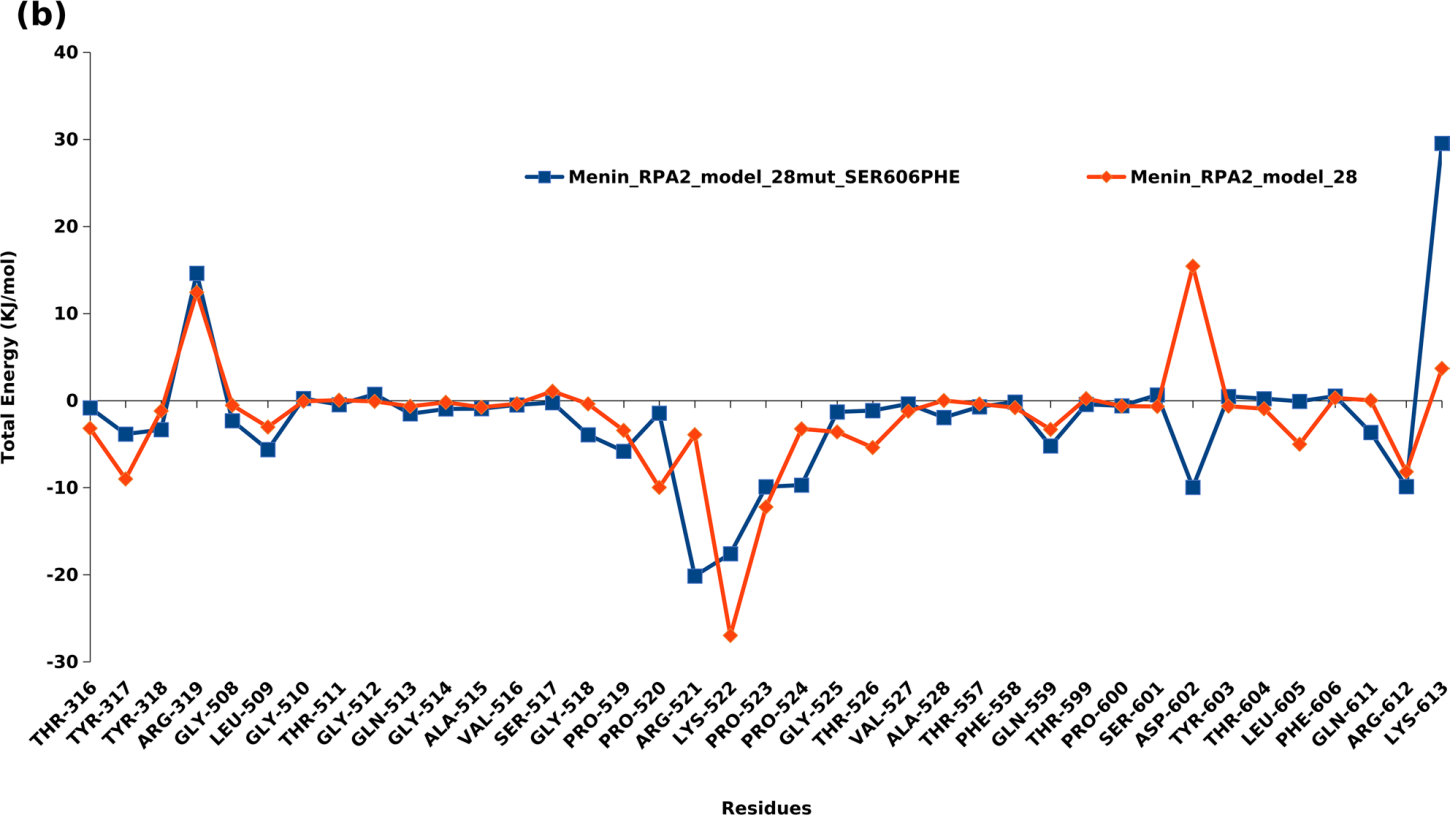
Energy decomposition analysis: depiction of per amino acid contribution to the total binding energy of wild and mutant type model 28 of Menin-RPA2.

After classical molecular dynamic simulation, the energy decomposition analysis was also performed to predict and quantify the interacted amino acids of Menin in RPA2 binding pockets of predicted model 8 and model 28 of wild type and mutant Menin-RPA2. Each BFE calculations were followed by EDA, which revealed the contribution of each residue in binding energy as shown in Figs. 5 and 6. In model 8, the RPA2 binding pocket in Menin was predicted to lie within amino acid position 69–74, 249–259, 260–262, 328–337, 371–383 and 606. However, in model 28, the RPA2 binding sites in Menin were composed of amino acids 316–319, 508–528, 557–606 and 611–613. The detailed position and amino acid involvement of Menin with RPA2 binding is illustrated in Figs. 5 and 6. Further, based on the per residue energy contribution to total binding energy of RPA2 binding to Menin, the specific amino acids of RPA2 involved in interaction were also predicted. In Model 8, the amino acid of RPA2 was predicted to lie within aa position 41–42, 46, 49, 63–65, 73–93, 105–108, 119, 127, 133–139, 142–125 and 171 (Supplementary Fig. S3). In Model 28, to amino acid of RPA2 involved in Menin interactions were lie within 41–46, 55, 58–69, 73, 76, 80–108, 127–145, 158, 169, and 171 (Supplementary Fig. S4). The detailed position and amino acid of RPA2 of model 8 and model 28 involved in binding with Menin were illustrated in Supplementary Figs. S3 and S4.

After constructing the Menin-RPA2 model with S606F point mutation in Menin, the contribution of highly fluctuating amino acids in terms of total energy (kJ/mol) were also investigated. In mutant Menin-RPA2 model 8, aspartate-70, Gylcine-74, Leucine-262, and Arginine-337 were mostly contributed to positive free energy, whereas, Proline 71, aspartate-253, Leucine 254, Histidine 255, Cysteine 334 and Glutamic acid 383 contributed to negative free energy i.e., stabilizing the PPi complex (Fig. 5). However, in mutant Menin-RPA2 model 28, Arginine-319, Glycine 510, Tyrosine 603, Threonine 604 and lysine 613 contributed to positive free energy and Leucine 509, Glycine 518, Proline 519, Arginine 521 and aspartate 602 contributed to negative binding free energy (Fig. 6).

### Configurational entropy calculations

**Figure 7:**
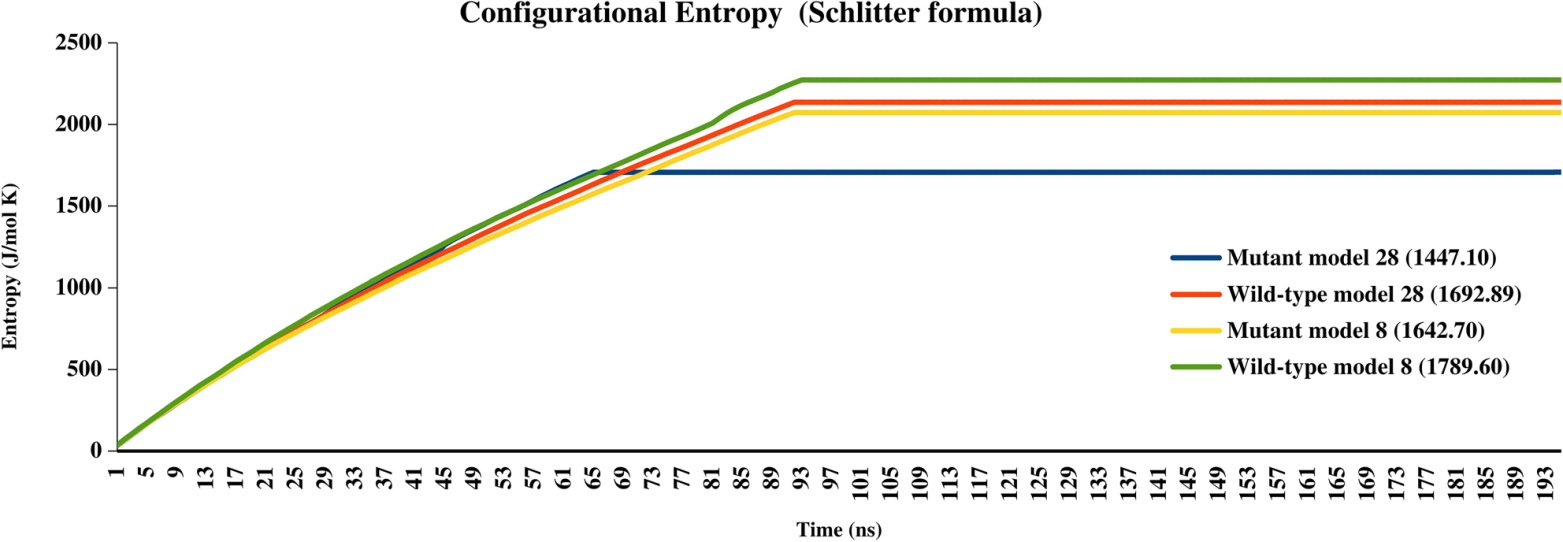
The cumulative configurational Entropy of Menin-RPA2 complex (Wild type & Mutant) during 200 ns.

We have also compared the cumulative configurational entropy per Cα-Cα atom of Menin-RPA2 complex for 200 ns as measure of extent of disorder after binding. As depicted in Fig. 7, the configurational entropy of mutant Menin-RPA2 model 28 was found to decreased to 1447.10 (J/mol K) at 61st ns as compared to wild type Menin-RPA2 complex (1642.70 J/mol K). Similar decrease of configurational entropy in mutant Menin-RPA2 model 8 (1642.70 J/mol K) was also measured in comparison with wild type Menin-RPA2 model 8 (1789.60 J/mol K). The trajectories during 200 ns simulations were shown in Fig. 7.

## Discussion

Menin plays a dominant role in the pathogenesis of multiple endocrine neoplasia type 1, embryonic development, and in normal regulation of cell growth and/or survival with its ability to regulate the activity of multiple transcription factors, such as Smad 3, JunD and NF-κB^21–24^. The identification of a menin-interacting protein like RPA2, with its association with DNA replication, recombination, repair, and transcription, provide a novel avenue of additional menin functions. In multiple endocrine neoplasia type 1, numerous mutations have been reported in *MEN1* gene that was known to disrupt the interaction of menin to RPA2 without affecting its binding to other factors such as Jun D. Previous studies on P12L, F144V and W183S mutations of RPA2 binding regions raise the possibility that RPA2 is important for the tumor suppressor activity of menin^21^. Previous studies have shown the interaction of RPA2 with menin for the maintenance of tumor suppressive activity^6^. However, the information corresponding to the complete structural model of menin with 612 amino acids were lacking, therefore predicting the complete structural model of menin is essential for investigating the binding sites of RPA2. Further, the information regarding the key interacting residues of menin with RPA2 is also insufficient.

Wet-lab experimentation alone is unable to provide readily identifiable values for the chemical phenomena that were involved in protein–protein interactions, further no quantum mechanical experimentation exists that we may use to measure interaction energies or chemical/physical components of protein–protein interactions. Therefore, the development of energy decomposition analysis (EDA) presents a novel approach to quantifying the contribution of binding site-specific amino acid. In this present study, we investigated the different interaction sites of RPA2 on Menin in order to pinpoint the specific amino acids contributing to the effective binding of RPA2-menin. Further, we have also analyzed the conformational perturbation in terms of binding free energy and energy decomposition analysis in mutated Menin protein.

As per the previous literature, the human menin crystal structures available on RCSB PDB (3U84) has highlighted that amino acid sequence length were up to 550 and rest were missing^25^. Therefore, in order to have a complete 3D structure of the Menin which is used in the present study, we constructed a full-chain model of human menin using FASTA sequence from UniProt (O00255). A recent study has also attempted to construct the 3D structure of menin using MODELLER 9.22^26^. However, in our study, we used the Swiss model to construct the 3D structure of menin, which is based on a hidden Markov model that allows more accurate 3D structure generation. In addition, a few studies have investigated the binding of menin with different transcription factors such as JunD and menin-mixed lineage leukemia factor 1 (MLL1) (21, 25 & 27); however, these studies were based on the selective sequence of the amino acid of menin. Thus, our study represents the more accurate and complete 3D model of Menin, which has the advantage over the experimental structures that served as a template in that it contains a complete chain that includes regions that are deleted or not visible in the experimental structures. In addition, AlphaFold is also an invaluable tool for predicting protein structures that could not be determined previously. Its programmatic approach and interactive visualizations allow users to gain insight into atomic coordinates, per-residue and pairwise model confidence estimates, and predicted alignment errors (28&29). Despite this significant advance in the field of protein structure prediction, the structure of menin was not properly modelled because the menin predicted in the AlphaFold database is not properly folded into the native 3D structure. However, regarding menin interacting protein RPA2, the full validated 3D structure was already available in previous studies, and the same structure was used to study the interaction of menin with RPA2^30^. To investigate the PPIs of menin and RP2, we performed computational protein–protein rigid docking and molecular dynamics simulations. Based on the results, 30 different menin-RPA2 interaction models were generated, and the best-fitting models of menin-RPA2 interaction were selected based on the ΔG energy. The binding energy-based prediction suggests effective interfacial residue–residue contacts in terms of predicting protein–protein binding^31^. Assessment of intermolecular contacts between the menin-RPA2 complex appears to be a better approach for predicting specific macromolecular arrangements. In the present study, three complexes (model 8, 28 and 9) were selected based on highest delta energy, which is − 74.89 kJ/mol, − 92.04 kJ/mol, and − 100.4 kJ/mol respectively. Among these three selected models, we have also assessed the time dependent (200 ns) average displacement of atoms during structural fluctuations in Menin-RPA2 complex. The averaged Cα-RMSD of model 8 of Menin-RPA2 were: 5.82 Å; model 9 of Menin-RPA2 were 6.52 Å and model 28 of Menin-RPA2 were 5.26 Å, therefore highlighting the different binding sites for RPA2 in Menin protein. The Cα-RMSD only entails the possibility of overall structural fluctuations during protein–protein complex formation in form of alignment scores.

In order to identify and validate the true positive binding site of RPA2 on Menin, binding free energy of different models of Menin-RPA2 were also evaluated. The binding free energy is a cumulative assessment of different terms associated with conformational entropy loss, hydrophobic contacts and hydrogen bonds or salt bridges^17,32,33^. The classical MD simulation followed by the MM/PBSA based energy calculation has greater robustness and more sensitive in terms of binding free energy calculation, trajectory evaluation as well as impact on configurational entropy^17,18^. g_mmpbs method is promising tool for studying the chemical relevance of energy components among different protein–protein interactions with good balance of speed and accuracy. After the analysis, model 8 of Menin-RPA2 exhibited most negative binding free energy of -205.624 kJ/mol, followed by model 28 of Menin-RPA2 with − 177.382 kJ/mol. However, Menin-RPA2 model 9 was excluded as it shows positive value of binding energy. Positive value of binding free energy depicts the unstable protein–protein complex formation, which may not be ideal for predicting the true binding site. Based on energy decomposition analysis, the RPA2 binding pocket in Menin were predicted to lie within amino acid position 69–74, 249–259, 260–262, 328–337, 371–383 and 606 in Model 8. Further, in model 28, the RPA2 binding sites in Menin were composed of amino acids 316–319, 508–528, 557–606 and 611–613. Based on our findings, Menin might have two binding pockets for RPA2 interactions. Also, we have identified Menin binding site on RPA2, which lie within amino acid position 41–171. This result is consistent with a previous study in which it was experimentally estimated that the sequences of the menin-binding region in RPA2 approximately map amino acids 43–171^6^. However, previous studies were unable to determine the exact amino acid residue of the RPA2 binding site in menin^6^. Thus, in this study, we determined the dual aspect, i.e., the menin binding site on RPA2 as well as the RPA2 binding site on menin.

**Figure 8:**
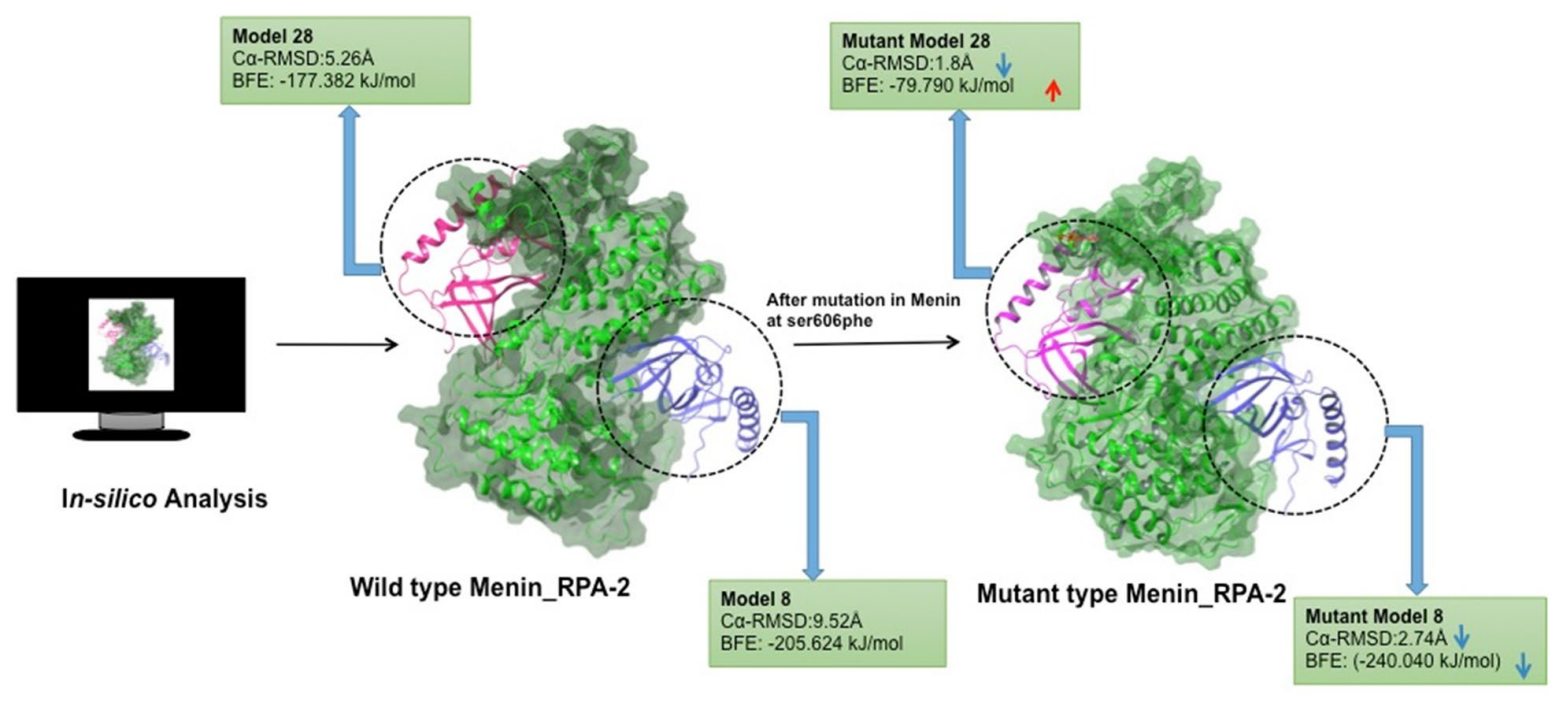
The schematic representation of in-silico analysis of Menin-RPA2 interactions and extended stability of Menin-RPA2 after S606F mutation in Menin.

In this study, we have also evaluated the detailed effect of S606F mutation in Menin on the menin-RPA2 interaction. Previous studies highlighted that S606F mutation in Menin could be pathological linked to development of multiple endocrine neoplasia type-1 syndrome^12^ that has already been validated in Indian PHPT populations. In a recent study, the computational significance of the mutation of the MEN1 gene on the structure of the native protein was also investigated using various computational tools, e.g., DUET web server and INPS3D^27,28,34,35^ The change of amino acid from serine to phenylalanine at 606 position leads to reduce Cα-RMSD in model 28 & model 8 of mutant Menin-RPA2. The reduction of Cα-RMSD in mutant model 28 and model 8 depicts the extended stability between Menin-RPA2 interactions. Similarly, binding free energy was also found to be decreased in model 8 and model 28 of mutant Menin-RPA2 interactions, depicted the increased binding affinity of RP2 with mutant Menin. Similarly, we have also evaluated the configurational entropy of Menin-RPA2 complex, calculated over the trajectories for 200 ns. Configurational entropy of bound state of two proteins calculates the degree of freedom between different complex that would overall predict the convergence of system^20,36^. In our study, we found significant decrease of configurational entropy in mutant menin-RPA2 complex during 200 ns simulation times. Previous studies^37,38^ have also reported the concept of extended stability in mutant protein, depicted through the decrease in RMSD value. Thus, the S606F mutation in Menin might lead reduction in Menin-RPA2 flexibility and backbone deviation, thereby facilitating very strong binding of Menin-RPA2. It’s noteworthy to state that different mutations have different phenotypic expressions. Our study has highlighted enhanced stability after S606F mutation in menin (Fig. 8). Interestingly, one study has reported the upregulation of RPA2 in hereditary breast cancer^10^. Chen, Chao-Chung, et al. reported that that the interaction of RPA2 with menin could inhibit menin-NF-κB interaction via a competitive binding mechanism. Based on our findings, the plausible explanation of the pathogenesis of multiple endocrine type-1 associated parathyroid adenoma could be conformational changes in S606F mutated menin that strongly stabilized the extended binding with RPA2, thereby hindering the binding of NF-κB.

### Limitations

Furthermore, evaluating RMSD, binding free energy and EDA in large mutation data of menin can further strengthened this study. The limitation of this study is lack of experimental validation for evaluating the concept of extended stability of the PPi after the incorporation of point mutation in menin.

## Conclusion

In the present study, MD simulations and configurational entropy calculations significantly highlighted the two different RPA2 binding sites on menin as well the functional effects of point mutation on Menin-RPA2 interactions. This is the first study to successfully elucidate the mechanism of extended stability of Menin-RPA2 complex induced by S606F point mutation. The energy decomposition analysis of wild type and mutant Menin-RPA2 complex identified specific amino acids involved in complex formations. Thus, identification of binding and stabilizing amino acids that are involved in menin-RPA2 interactions have provided insights into the functioning of menin & RPA2 complex which will facilitate the designing of targeted treatment of multiple tumour types, RPA2 related pathologies and can also expanded experimentally for greater insights.

## Supplementary Information

Supplementary Information.

## Data Availability

All data generated or analyzed during this study are included in this published article and its supplementary information files.
