# Supplementary material for: Investigating the novel-binding site of RPA2 on Menin and predicting the effect of point mutation of Menin through protein–protein interactions
Source: Sci Rep. 2023 Jun 8;13:9337. doi: 10.1038/s41598-023-35599-2 (PMC10250348; doi:10.1038/s41598-023-35599-2)
Supplement: Supplementary file 1 — Supplementary Information. [file 41598_2023_35599_MOESM1_ESM.docx]

**Supplementary figures**

**Supplementary figure 1 (S1):** The Ramachandran plot of Menin protein.

**
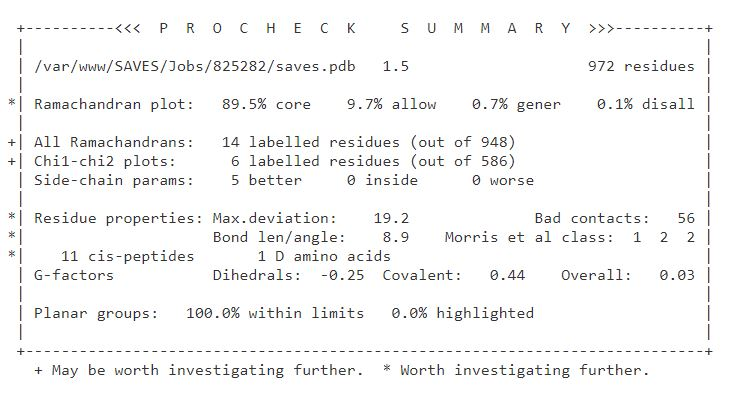
**

**Supplementary figure 2 (S2):** The validation of RPA-2 using of PROCHECK tool.

**
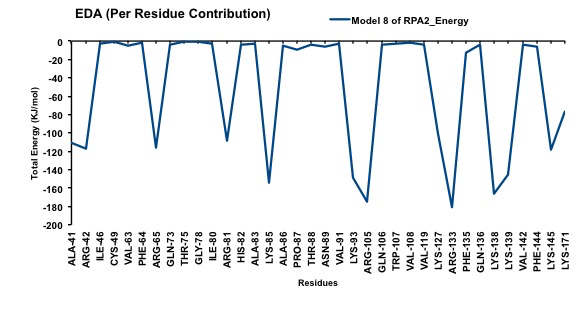
**

**Supplementary figure 3 (S3):** Energy decomposition analysis: Depiction of per amino acid contribution to the total binding energy of RPA2 in model 8

**
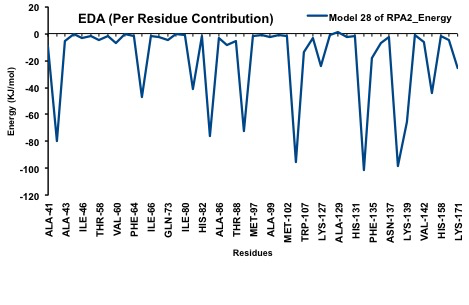
**

**Supplementary figure 4 (S4):** Energy decomposition analysis: Depiction of per amino acid contribution to the total binding energy of RPA2 in model 28

**Supplementary Information**

**Annexure 1:**

**FASTA SEQUENCE OF MENIN:**

>sp|O00255|MEN1_HUMAN Menin OS=Homo sapiens OX=9606 GN=MEN1 PE=1 SV=4 MGLKAAQKTLFPLRSIDDVVRLFAAELGREEPDLVLLSLVLGFVEHFLAVNRVIPTNVPE LTFQPSPAPDPPGGLTYFPVADLSIIAALYARFTAQIRGAVDLSLYPREGGVSSRELVKK VSDVIWNSLSRSYFKDRAHIQSLFSFITGWSPVGTKLDSSGVAFAVVGACQALGLRDVHL ALSEDHAWVVFGPNGEQTAEVTWHGKGNEDRRGQTVNAGVAERSWLYLKGSYMRCDRKME VAFMVCAINPSIDLHTDSLELLQLQQKLLWLLYDLGHLERYPMALGNLADLEELEPTPGR PDPLTLYHKGIASAKTYYRDEHIYPYMYLAGYHCRNRNVREALQAWADTATVIQDYNYCR EDEEIYKEFFEVANDVIPNLLKEAASLLEAGEERPGEQSQGTQSQGSALQDPECFAHLLR FYDGICKWEEGSPTPVLHVGWATFLVQSLGRFEGQVRQKVRIVSREAEAAEAEEPWGEEA REGRRRGPRRESKPEEPPPPKKPALDKGLGTGQGAVSGPPRKPPGTVAGTARGPEGGSTA QVPAPTASPPPEGPVLTFQSEKMKGMKELLVATKINSSAIKLQLTAQSQVQMKKQKVSTP SDYTLSFLKRQRKGL

**FASTA SEQUENCE OF RPA2 :**

>2PI2_1|Chains A, B, C, D|Replication protein A 32 kDa subunit|Homo sapiens (9606) MWNSGFESYGSSSYGGAGGYTQSPGGFGSPAPSQAEKKSRARAQHIVPCTISQLLSATLVDEVFRIGNVEISQVTIVGIIRHAEKAPTNIVYKIDDMTAAPMDVRQWVDTDDTSSENTVVPPETYVKVAGHLRSFQNKKSLVAFKIMPLEDMNEFTTHILEVINAHMVLSKANSQPSAGRAPISNPGMSEAGNFGGNSFMPANGLTVAQNQVLNLIKACPRPEGLNFQDLKNQLKHMSVSSIKQAVDFLSNEGHIYSTVDDDHFKSTDAE
